# Single cell Raman spectroscopy to identify different stages of proliferating human hepatocytes for cell therapy

**DOI:** 10.1186/s13287-021-02619-9

**Published:** 2021-10-30

**Authors:** Chen Ma, Ludi Zhang, Ting He, Huiying Cao, Xiongzhao Ren, Chenhui Ma, Jiale Yang, Ruimin Huang, Guoyu Pan

**Affiliations:** 1 Shanghai Institute of Materia Medica, Chinese Academy of Sciences, Shanghai, 201203 China; 2 University of Chinese Academy of Sciences, Beijing, 100049 China; 3 State Key Laboratory of Cell Biology, CAS Center for Excellence in Molecular Cell Science, Shanghai Institute of Biochemistry and Cell Biology, Chinese Academy of Sciences, Shanghai, China; 4 University of Chinese Academy of Science, Beijing, China; 5 School of Pharmaceutical Sciences, Nanjing Tech University, Nanjing, China; 6 State Key Laboratory of Cell Biology, CAS Center for Excellence in Molecular Cell Science, Institute of Biochemistry and Cell Biology, University of Chinese Academy of Sciences, Chinese Academy of Sciences, Shanghai, 200031 China

**Keywords:** Raman spectroscopy, Cell therapy, Identification, Proliferating human hepatocytes, Dedifferentiation

## Abstract

**Background:**

Cell therapy provides hope for treatment of advanced liver failure. Proliferating human hepatocytes (ProliHHs) were derived from primary human hepatocytes (PHH) and as potential alternative for cell therapy in liver diseases. Due to the continuous decline of mature hepatic genes and increase of progenitor like genes during ProliHHs expanding, it is challenge to monitor the critical changes of the whole process. Raman microspectroscopy is a noninvasive, label free analytical technique with high sensitivity capacity. In this study, we evaluated the potential and feasibility to identify ProliHHs from PHH with Raman spectroscopy.

**Methods:**

Raman spectra were collected at least 600 single spectrum for PHH and ProliHHs at different stages (Passage 1 to Passage 4). Linear discriminant analysis and a two-layer machine learning model were used to analyze the Raman spectroscopy data. Significant differences in Raman bands were validated by the associated conventional kits.

**Results:**

Linear discriminant analysis successfully classified ProliHHs at different stages and PHH. A two-layer machine learning model was established and the overall accuracy was at 84.6%. Significant differences in Raman bands have been found within different ProliHHs cell groups, especially changes at 1003 cm^−1^, 1206 cm^−1^ and 1440 cm^−1^. These changes were linked with reactive oxygen species, hydroxyproline and triglyceride levels in ProliHHs, and the hypothesis were consistent with the corresponding assay results.

**Conclusions:**

In brief, Raman spectroscopy was successfully employed to identify different stages of ProliHHs during dedifferentiation process. The approach can simultaneously trace multiple changes of cellular components from somatic cells to progenitor cells.

**Supplementary Information:**

The online version contains supplementary material available at 10.1186/s13287-021-02619-9.

## Background

Liver disease is widely concerned among global researchers with 2 million deaths every year [1]. A lot of liver diseases may lead to liver cirrhosis and cancer, which may trigger liver failure [2]. Orthotopic liver transplantation is currently the final solution for liver failure. Nevertheless, the healthy liver doners are very limited. The emergence of cell therapy presents new opportunities for multiple liver diseases. It was reported that hepatocytes transplantation could relieve or even cure liver diseases [3–5]. Because primary human hepatocytes (PHH) are unable to proliferate in vitro, the availability of qualified PHH in clinic become the bottleneck of hepatocytes transplantation.

During the past decades, multiple cell sources have been investigated for liver transplantation as alternative of PHH. For example, fetal liver progenitors, adult human liver stem cells, hematopoietic stem cells, mesenchymal stem cells, human pluripotent stem cells, and induced pluripotent stem cells [6–13]. It was reported that rat fetal liver progenitor cells could differentiate into hepatocytes and reduce fibrotic activity after transplantation. Its efficacy is better than rat primary hepatocytes [14]. However, it is unfeasible to obtain enough human liver progenitor cells in clinic, not to mention conduct any quality control.

Zhang et al. found Wnt3a appeared to be essential to the initiation of the proliferation of PHH. The significant upregulation of Wnt target genes lead to the proliferation of PHH in vitro, and the proliferating human hepatocytes (ProliHHs) showed both hepatocyte and progenitor features and could be reverted to mature phenotypes and rescue liver failure mice with high efficiency [15]. ProliHHs mature hepatic markers will gradually decrease while progenitor associated markers will increase during the passage. The progenitor-like cells could be restored to mature status (matured ProliHHs) in 3D culture process. Qiao et al. indicated that the matured ProliHHs had comparable metabolic capacities and biliary excretion capacity [16]. Nevertheless, maturation of ProliHHs may not be necessary for cell therapy in liver diseases, liver progenitor cells could be a better option for cell transplantation.

Because the hepatocytes proliferating and dedifferentiation to ProliHHs is a continuous process, a quick and non-invasive quality control method is critical to ensure the quality of harvested ProliHHs for liver transplantation. Conventional techniques, such as RT-qPCR and Western blotting need a lot of cells and are time-consuming. Theoretically, flow cytometric could precisely measure the quantities of antigens in each cell, however, they must be labelled with specific antibodies and/or fluorescently labelled microbeads [17].

Raman microspectroscopy has unique advantages in the analysis of multiple biological substances [18, 19]. The inelastic scattered light is collected to reflect biochemical molecules. It is nondestructive, noninvasive, label free, high sensitivity and resolution [20–22]. A single spectrum could be obtained in a few seconds which include information about multiple substances such as nucleic acids, proteins, lipids, saccharides and so on. In addition, the subcellular structures can be visualized by Raman image as well. For example, the accumulation of lipid droplets was observed in mice hepatocytes during nonalcoholic fatty liver disease (NAFLD) development [23]. Furthermore, Raman spectroscopy allows to monitor the various components at the single cell level which is suitable to provide cell heterogeneous information [24]. The analytical technique has been used to monitor cell metabolism, cell sorting, disease diagnosis and other related fields [25–31].

In this study, an established in-house protocol was employed to help PHH dedifferentiated into ProliHHs. PHH is defined as ProliHHs passage 0. ProliHHs at different stages (from passage 0 to passage 4, the later generation is closer to progenitor-like cells) were utilized to collect Raman spectra. The supervised linear discriminant analysis (LDA) was able to reduce the high dimensionality of data and distinguish three clearly clusters. Furthermore, the significant difference bands were semi-quantified and attributed to the corresponding biomolecules. The Raman bands related to phenylalanine, hydroxyproline and lipids were chosen as representative biomarkers to verify the reliability of Raman. reactive oxygen species (ROS), hydroxyproline and triglyceride levels were measured from PHH to ProliHHs (P1 and P4). The results of the two methods are consistent. Finally, the machine learning model was successfully established and used to rapidly identify cells stages with an overall accuracy of 84.6%. Importantly, it is the first time to indication that the changes of biochemical components during the dedifferentiation process from somatic cells (primary human hepatocytes) to progenitor like cells (ProliHHs) by Raman spectroscopy.

## Methods

### Cell culture

The primary human hepatocytes (Lot: 005 and Lot: 201678901, Novabiosis) were used to induced ProliHHs in the study. Cryopreserved PHH were thawed and seeded into Matrigel (Corning)-coated 6-well culture plates at 200,000 viable cells per well. After 6 h, cell medium was replaced by HM as previously published protocols. HM was mixed by 500 ml Advanced DMEM/F-12 (Life Technologies), 1 $\times$ N2 supplement 100 (Life Technologies), 1 $\times$ B27 Supplement 50 minus vitamin A (Life Technologies), 1 mM N-acetyl-cysteine (Sigma-Aldrich), 10 mM Nicotinamide (Solarbio), 2 ng/ml Recombinant humanFGF10 (Peprotech), 50 ng/ml Recombinant human EGF (Peprotech), 25 ng/ml Recombinant human HGF (Peprotech), 10 nM Human [Leu15]-gastrin I (Sigma-Aldrich), 5uM A 83–01 (Tocris Bioscience), 10uM Rho kinase inhibitor Y-27632 (Selleck), 50 ng/ml Wnt3a protein (stemimmune LLC), 1% Fetal bovine serum (Ausbian) [15]. The cell culture medium was changed every 3 days. After 7 days, PHH were successfully induced ProliHHs by HM and 2% hypoxic culture. Cells were washed with PBS and trypsinized for passaging when they reached 90% confluence. ProliHHs were incubated in 37℃, hypoxia (5% CO_2_, 2% O_2_) incubator. ProliHHs (P0, P1 and P4) morphology were performed by phase contrast microscopy after cultured 1 day. HepG2 cells were obtained from ATCC. 293FT cells were provided by Professor Lijian, Hui (State Key Laboratory of Cell Biology, CAS Center for Excellence in Molecular Cell Science, Shanghai Institute of Biochemistry and Cell Biology, Chinese Academy of Sciences, University of Chinese Academy of Science). Hepatoblast cells were provided by Professor Xin Cheng (State Key Laboratory of Cell Biology, CAS Center for Excellence in Molecular Cell Science, Institute of Biochemistry and Cell Biology, University of Chinese Academy of Sciences, Chinese Academy of Sciences). Primary rat hepatocytes were isolated from SD rats according to previous protocol [32].

### RT-qPCR analysis

ProliHHs (P0, P1 and P4) were maintained on Matrigel-coated 12-well plates at 250,000 viable cells per well. After 24 h, samples were collected by TRIzol reagent (Life Technology) and the EZ-10 Spin column & Collection Tubes (Sangon Biotech). 500 ng RNA was reversely transcribed to cDNA using Hifair® III 1st Strand cDNA Synthesis SuperMix (Yeasen Biotech). cDNA was amplified by Hieff® qPCR SYBR Green Master Mix (Yeasen Biotech) on the Applied Biosystems 7500 Fast real-time PCR System (Thermo Fisher Scientific). Primers sequences were listed in Table S1. The relative mRNA levels were normalized by GAPDH. Each sample was performed in 3 replicates. GraphPad Prism 8.0 software was used to analyze data. The results represent means ± SD. One-way ANOVA was used for statistical analysis, ns *p* ≥ 0.05, * *p* < 0.05, ** *p* < 0.01, *** *p* < 0.001, **** *p* < 0.0001.

### Reactive oxygen species measurements

ProliHHs (P0, P1 and P4) were seeded on Matrigel-coated 96-well plates at 50,000 viable cells per well and incubated overnight. Cells were washed in serum-free medium and treated with 10 mol$\upmu$/L DCFH-DA (Beyotime Biotechnology) for 20 min at 37℃. Then cells were washed 3 times in serum-free medium and measured fluorescence intensity at 488 nm excitation and 525 nm emission by the automatic microplate reader (Biotek).

### Triglyceride measurements

ProliHHs (P0, P1 and P4) were cultured on Matrigel-coated 96-well plates at 50,000 viable cells per well. Cells were washed with PBS and removed supernatant. Then, 10 μl RIPA (Beyotime Biotechnology) was added into cells per well for 10 min. Lysate was determined protein content by Take3 Micrometer plate (BioTek) and TG levels by Triglyceride assay kit (Nanjing Jiancheng Bioengineering Institute) according to manufacturer’s instructions.

### Hydroxyproline measurements

ProliHHs (P0, P1 and P4) were maintained on Matrigel-coated 12-well plates at 1000,000 viable cells per well. After 24 h, the supernatant was collected and the hydroxyproline concentration was measured by Hydroxyproline assay kit (Nanjing Jiancheng Bioengineering Institute) according to manufacturer’s instructions.

### Raman spectroscopy measurements

ProliHHs (P0, P1 and P4) were seeded on 8-Well Chamber Raman Scattering Microslide (D-BAND) as cell density 100,000 cells/ml for 24 h. Cells were washed with PBS, fixed with 4% paraformaldehyde (Beyotime Biotechnology) for 15 min, washed 3 times with sterile water. Then, the cells on Raman Scattering Microslide were air dried. Raman measurements were performed with a confocal Raman imaging system (alpha 300 R, WITec). The system was included in a 532 nm laser, 1800 grooves/mm grating (BLZ = 500 nm) and a CCD camera. Raman spectra (from 300 to 1800 cm^−1^) were collected by a 100 $\times$ objective (N.A. = 0.9) with laser power 9 mW, integration time 10 s and accumulation number 1. Calibration was performed using a silicon plate with its unique peak located at 520.7 cm^−1^. Raman spectra for each cell were randomly acquired within the cytoplasm (*n* = 5) and on the periphery (*n* = 5), respectively, based on the brightfield photo. A representative image with different focusing locations of laser was shown in Additional file 1: Figure S1. For each cell type, Raman spectra (*n* = 600 in total) were acquired from 20 single cells per batch and 3 replicate batches. All spectra were normalized with subtraction of cosmic ray, baseline correction and area normalization.

### Raman data analysis

All Raman data analysis were done under an R 3.6.3 environment with inhouse scripts. Linear discriminant analysis (LDA) and Principal component analysis (PCA)were used to reduce data dimensions and visualize classification. The Raman bands area were integrated to semiquantitative associated with biomolecules. The results represent median. The Student's t-test was used for statistical analysis, ns *p* ≥ 0.05, * *p* < 0.05, ** *p* < 0.01, *** *p* < 0.001, **** *p* < 0.0001. Machine learning models were applied to build databases to rapidly identify cells. 75% data was used as a training set to establish model, 25% as a test set to evaluate model. It was used to build model by tenfold cross validation with five repetitions. Primarily, single classifiers were measured by k-nearest neighbor, linear discriminant analysis, partial least-squares regression, linear support vector machine, radial basis function kernel support vector machine and random forest. In order to improve the accuracy of prediction, total of single classifiers were stacked together forming a two-layer model by GBM algorithm. The characteristics of the second layer is the single model results (KNN, LDA, PLS, Linear-SVM, RBF-SVM and RF), according to Hsu et al. protocol [33].

## Results

### Generation and characterization of ProliHHs

**Fig. 1:**
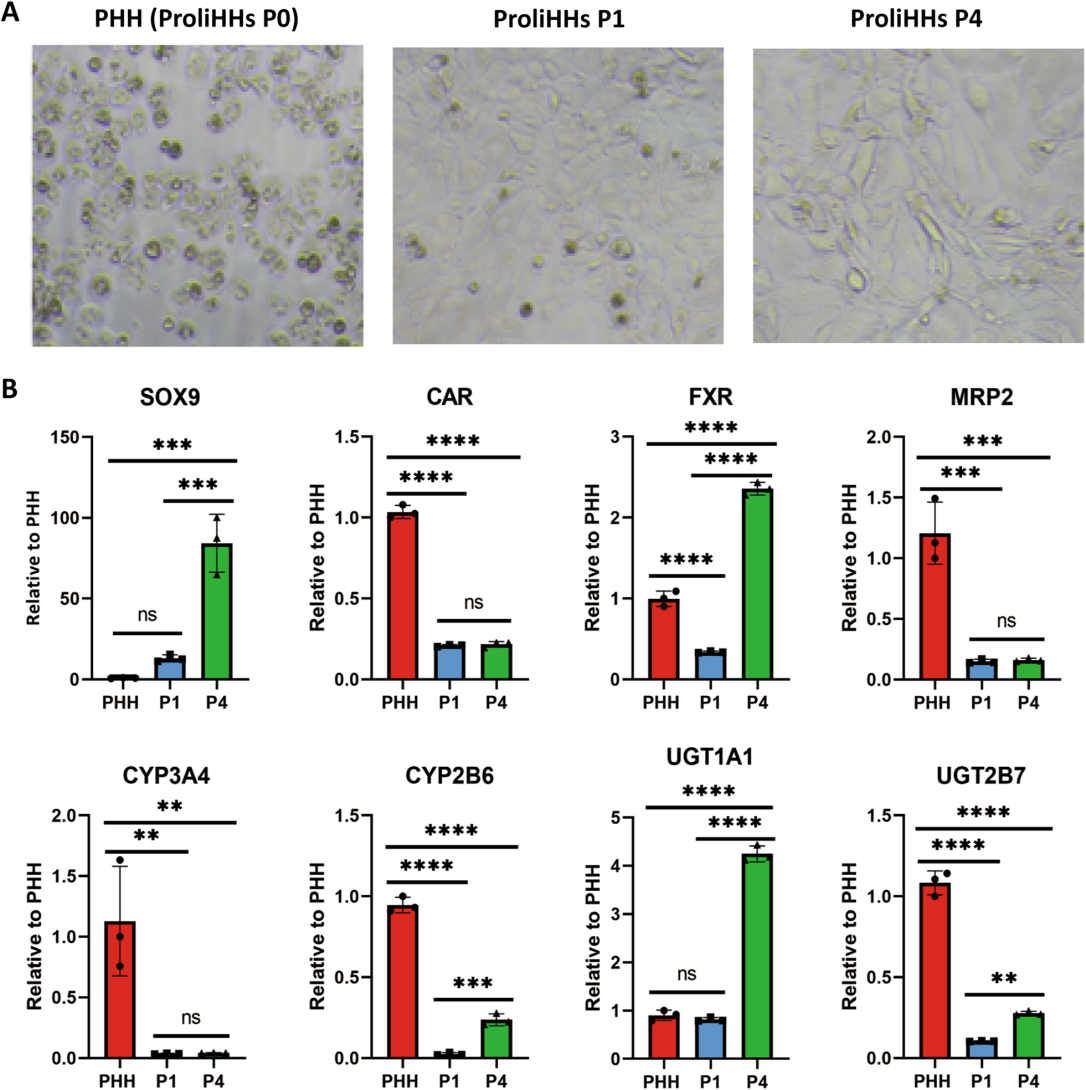
ProliHHs were derived from PHH (Lot:005) and identified by mRNA. **a** Microscopic imaging of PHH, ProliHHs P1 and P4 (magnification, × 10). **b** The mRNA expressions of biomarkers relative to hepatocytes and progenitor cells in PHH, ProliHHs P1 and P4. The results represent means ± SD, ns *p* $\ge$ 0.05, * *p* < 0.05, ** *p* < 0.01, *** *p* < 0.001, **** *p* < 0.0001. (PHH: primary human hepatocytes, ProliHHs: proliferating human hepatocytes, P1: passage 1, P4: passage 4)

Previous researches showed that ProliHHs were bi-phenotypical cells which expressed genes of mature hepatocytes and liver progenitors [15]. To some extent, ProliHHs could replace primary human hepatocytes for drug safety evaluation and cell therapy [16]. However, there were a lot of gene expression and function differences among PHH and different passages of ProliHHs. Thus, a quick and simple approach to identify cells stages is urgently needed. Here, PHH were induced to ProliHHs by HM medium and hypoxia cultured for 7d. PHH could expand more than 250 folds to ProliHHs at P4. PHH and P1were polygonal, P4 partly were long strips. (Fig. 1a). SOX9, as a biomarker for liver progenitor cells, mRNA expression was gradually increased from PHH to ProliHHs (P1 and P4). The nuclear receptors FXR and CAR, mature hepatic genes, expressed in PHH, P1 and P4. Interestingly, the FXR level of P4 was higher than PHH. The efflux transporter MRP2 mRNA level was downregulated both P1 and P4 relative to PHH. Although ProliHHs expressions of Phase I drug metabolizing enzymes, including CYP3A4 and CYP2B6, were lower than PHH. The CYP2B6 gene of P4 showed nearly 1/3 recovery compared to PHH. Phase II drug metabolizing enzymes, such as UGT1A1 and UGT2B7, were also expression of P1 and P4. In particular, the UGT1A1 level was significantly higher than PHH (Fig. 1b). In brief, from gene expression perspective, ProliHHs only maintained part of drug metabolic and transport genes, the cells expressed more and more liver progenitor biomarkers from P1 to P4.

### Raman spectroscopy and classification analysis

**Fig. 2:**
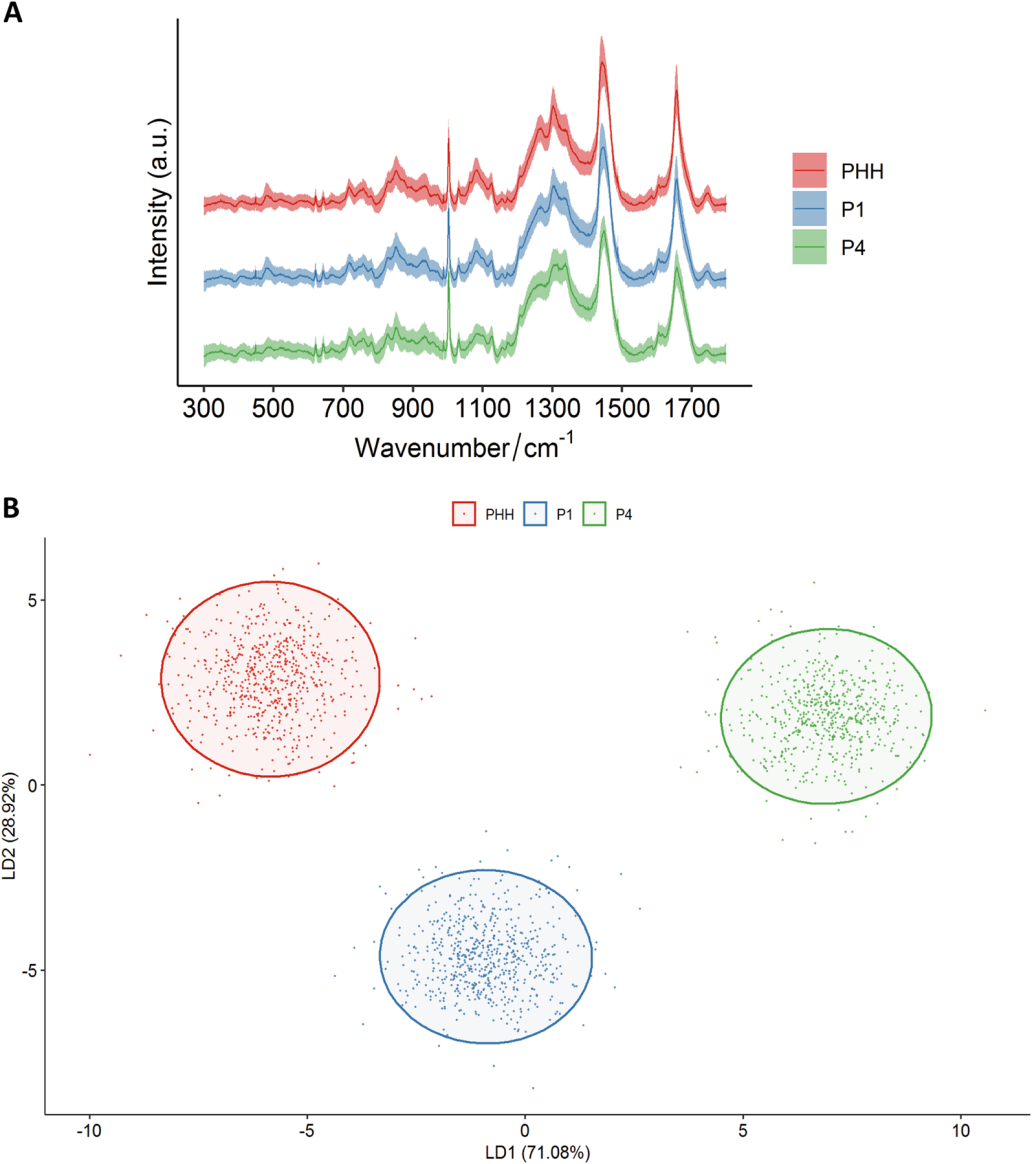
Raman spectroscopy and classification analysis for PHH (Lot:005), ProliHHs P1 and P4. **a** The averaged spectra (*n* = 1850) collected by PHH (*n* = 620), P1 (*n* = 624) and P4 (*n* = 606) on fingerprint region. **b** Linear discriminant analysis clearly distinguished three cell groups. (The red, blue, and green colors represent PHH, ProliHHs P1 and P4 cells, respectively. PHH: primary human hepatocytes, ProliHHs: proliferating human hepatocytes, P1: passage 1, P4: passage 4)

Although the mRNA and protein levels could characterize the differences between PHH and different passages of ProliHHs, those methods cost much time while get limited information. Therefore, we evaluated the potential of collected Raman spectroscopy to monitor cell to cell heterogeneity. PHH, ProliHHs P1 and P4 were respectively examined at least 600 single spectrum (Table 1). The fingerprint region, including more than 90% of the cellular peaks, was collected to identify cell stages [34]. The averaged spectra of each type cell were showed in Fig. 2a. Principal component analysis (Additional file 1: Fig. S1) and linear discriminant analysis (Fig. 2b) were used to reduce dimension and highlight the spectral signatures on different cell stages. One Raman sample was represented as a point, at least 600 samples to reflect each type cells in different figures. Obviously, LDA was able to discriminate cell populations better than PCA. The significant wavenumbers in LD1 and LD2 contributed to differences among three groups were provided in (Additional file 1: Fig. S2). This suggests the ability of Raman spectroscopy to classify cells.

**Table 1:** Numbers of Raman spectra acquired from PHH, ProliHHs P1 and P4

Group	Number of spectra
PHH	620
ProliHHs P1	624
ProliHHs P4	606

### Machine learning models

In addition to extract significant wavenumbers, it is suitable to establish Raman dataset by machine learning models. The entire Raman spectral (1850) was randomly divided into two parts, 75% to establish the training set, 25% to verify the model. First, the single model KNN, LDA, PLS, Linear-SVM, RBF-SVM and RF were respectively constructed to classify by tenfold cross-validation with five repetitions and the corresponding parameters were showed in Additional file 2: Table S2. The overall accuracy was 83.8% in Linear-SVM model. Then, the fitted results of above six models were stacked to form a two-layer machine learning model, which improved the prediction accuracy to 84.6% (Table 2). The sensitivities were 75.6%, 88.7%, 89.7% and specificity were 90.2%, 95.5%, 81.2% for PHH, P1 and P4.

**Table 2:** Machine learning by stacked (KNN, LDA, PLS, Linear-SVM, RBF-SVM, RF) model to identify cells. Overall accuracy at 84.6%

	Reference		
	P1	P4	PHH
Model prediction			
ProliHHs P1	118	14	16
ProliHHs P4	14	134	0
PHH	24	3	139
Sensitivity (%)	75.6	88.7	89.7
Specificity (%)	90.2	95.5	81.2

### Potential biomarkers of cell identification

**Fig. 3:**
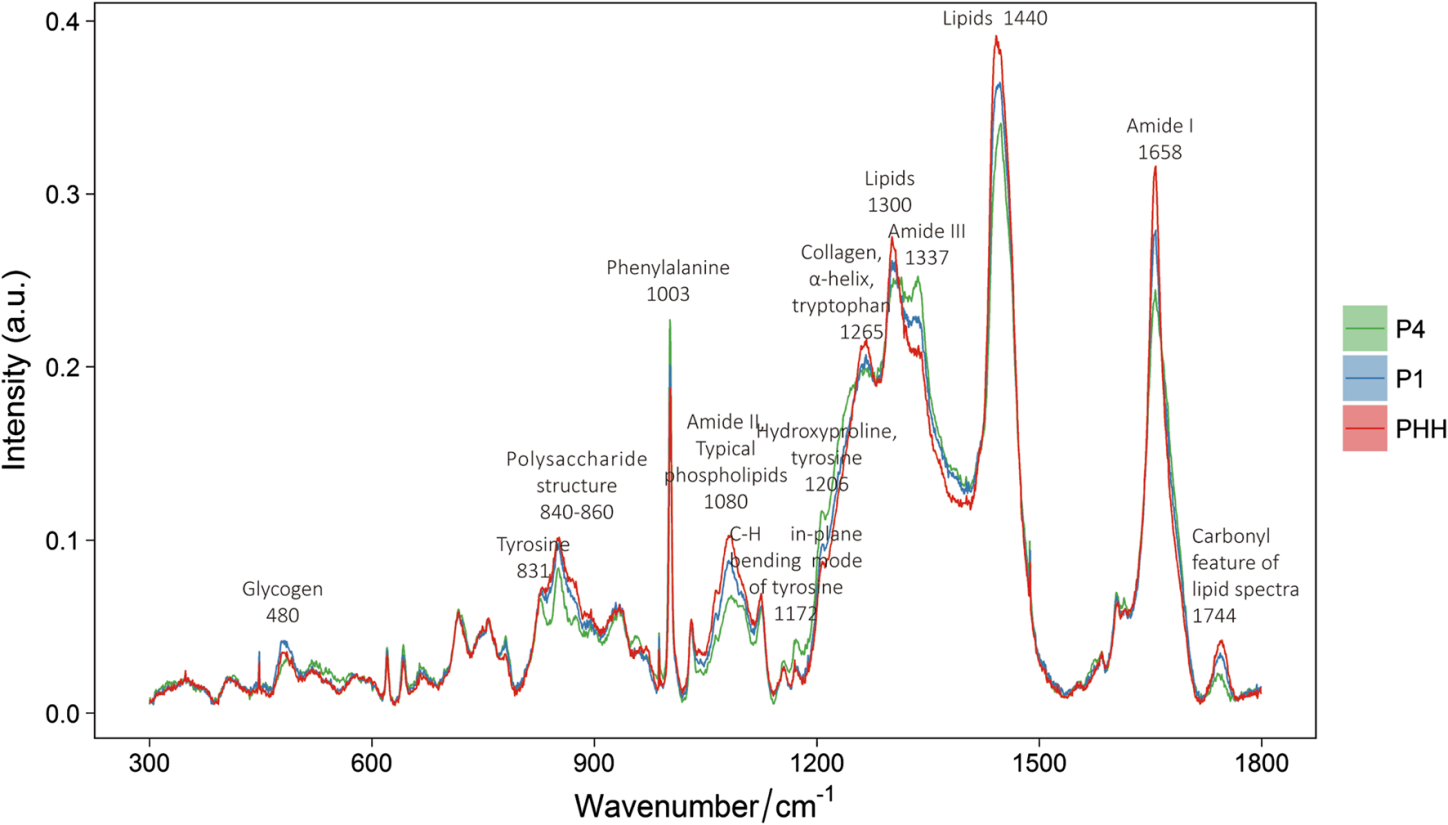
The biochemical molecules represented by the specific Raman bands in the average spectral (Lot:005)

**Fig. 4:**
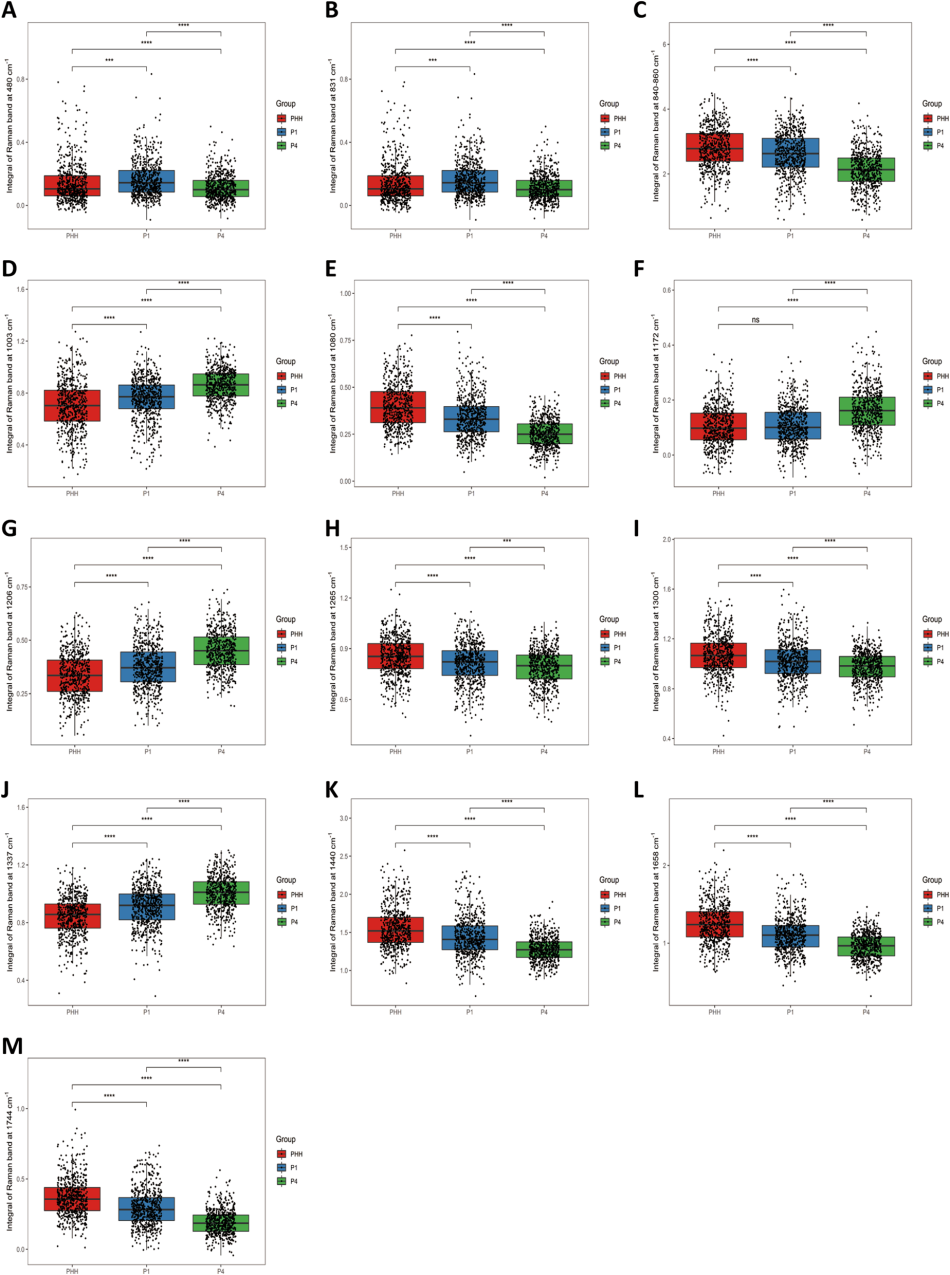
The peak area were semi-quantitative to compare differences of the specific Raman bands **a** 480 ${\mathrm{cm}}^{-1}$ (glycogen), **b** 831 ${\mathrm{cm}}^{-1}$ (tyrosine), **c** 840–860 ${cm}^{-1}$(polysaccharide structure), **d** 1003 ${\mathrm{cm}}^{-1}$ (phenylalanine), **e** 1080 ${\mathrm{cm}}^{-1}$ (amide II, typical phospholipid), **f** 1172 ${\mathrm{cm}}^{-1}$ (C–H in-plane bending mode of tyrosine), **g** 1206 ${\mathrm{cm}}^{-1}$ (hydroxyproline, tyrosine), **h** 1265 ${\mathrm{cm}}^{-1}$ (α-helix, collagen, tryptophan), **i** 1300 ${\mathrm{cm}}^{-1}$ (lipids), **j** 1337 ${\mathrm{cm}}^{-1}$ (amide III), **k** 1440 ${\mathrm{cm}}^{-1}$ (lipids), **l** 1658 ${\mathrm{cm}}^{-1}$ (amide I), **m** 1744 ${\mathrm{cm}}^{-1}$ (carbonyl feature of lipid spectra) in PHH (Lot:005), ProliHHs P1 and P4. The results represent median, ns *p* $\ge$ 0.05, * *p* < 0.05, ** *p* < 0.01, *** *p* < 0.001, **** *p* < 0.0001 (PHH: primary human hepatocytes, ProliHHs: proliferating human hepatocytes, P1: passage 1, P4: passage 4)

There were numbers of differences in Raman bands which may reflect cell biochemical components changes from PHH to different stages of ProliHHs (P1 and P4) (Fig. 3). The results suggested significant differences among spectral bands of cell clusters located at 480 cm^−1^ (glycogen), 831 cm^−1^ (tyrosine), 840–860 cm^−1^ (polysaccharide), 1003 cm^−1^ (phenylalanine), 1080 cm^−1^ (amide II, typical phospholipids), 1172 cm^−1^ (C–H in plane bending mode of tyrosine), 1206 cm^−1^ (hydroxyproline),1265 cm^−1^ ($\alpha$-helix, collagen, tryptophan), 1300 cm^−1^ (lipids), 1337 cm^−1^ (Amide III), 1440 cm^−1^ (CH_2_ and CH_3_ formation vibrations of lipids), 1658 cm^−1^ (Amide I of proteins), 1744 cm^−1^ (carbonyl feature of lipid spectra) respectively. To further quantify the changes in 3 groups, relative Raman bands area were integrated (Fig. 4a–m). The peak at 480 cm^−1^ (*p* $\le$ 0.001) and 831 cm^−1^ (*p* $\le$ 0.001) were significant difference between PHH and ProliHHs (P1 and P4). The Raman bands area at 840–860 cm^−1^ (*p* $\le$ 0.0001), 1080 cm^−1^ (*p* $\le$ 0.0001), 1265 cm^−1^ (*p* $\le$ 0.001), 1300 cm^−1^ (*p* $\le$ 0.0001), 1440 cm^−1^ (*p* $\le$ 0.0001), 1658 cm^−1^ (*p* $\le$ 0.0001) and 1744 cm^−1^(*p* $\le$ 0.0001) were significantly decreased following ProliHHs derived and passaged. In contrast, the Raman bands area at 1003 cm^−1^ (*p* $\le$ 0.0001), 1206 cm^−1^ (*p* $\le$ 0.0001), 1337 cm^−1^ (*p* $\le$ 0.0001) were significantly increased. Although the band at 1172 cm^−1^ was no change between PHH and P1, P4 was higher than the others.

**Fig. 5:**
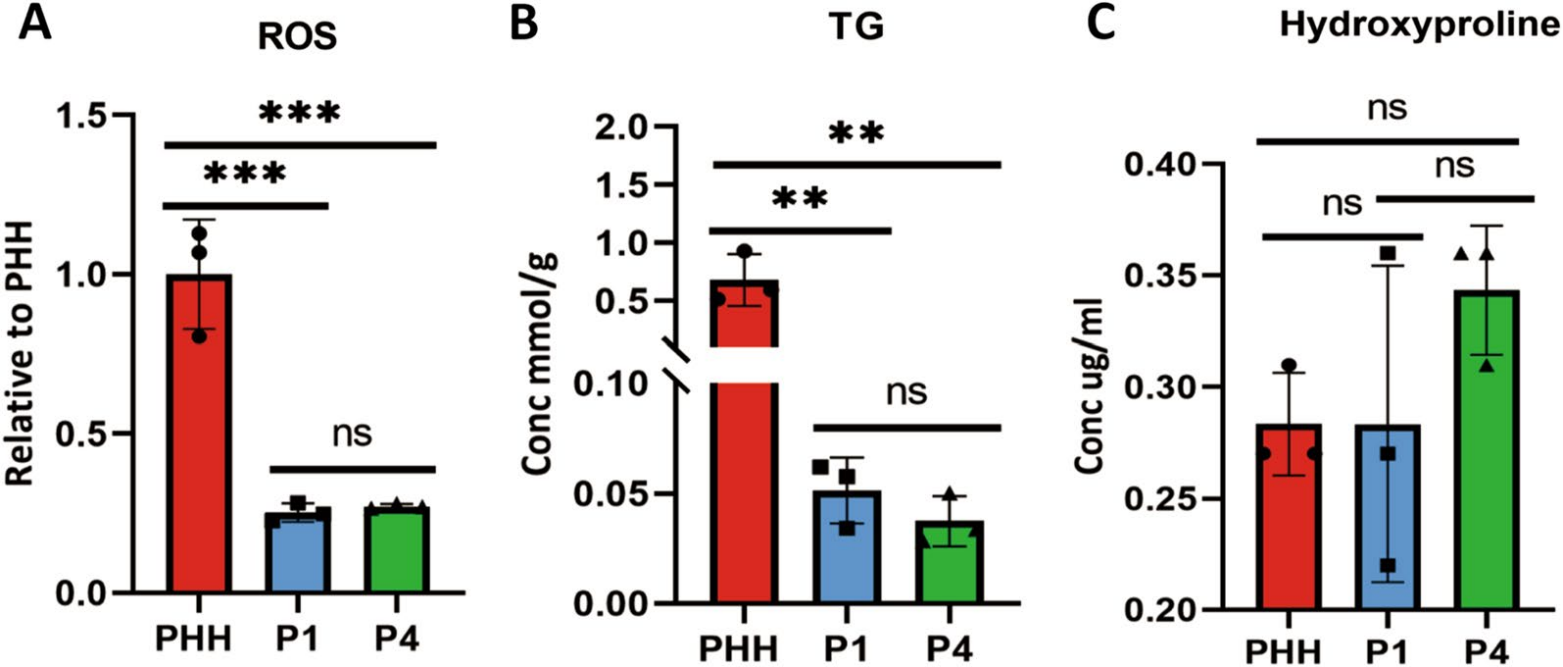
The potential biomarkers to identify PHH (Lot:005), P1 and P4. **a** Reactive oxygen species (ROS) levels decreased **b** triglyceride (TG) concentration decreased and **c** hydroxyproline concentration increased from PHH to ProliHHs (P1 and P4). The results represent means ± SD, ns *p* $\ge$ 0.05, * *p* < 0.05, ** *p* < 0.01, *** *p* < 0.001, **** *p* < 0.0001 (PHH: primary human hepatocytes, ProliHHs: proliferating human hepatocytes, P1: passage 1, P4: passage 4)

The Raman bands changes at 1003 cm^−1^, 1206 cm^−1^ and 1440 cm^−1^ were identified with reactive oxygen species, hydroxyproline and triglyceride levels by the corresponding kit. The ROS levels significant down regulated from PHH to ProliHHs (P1 and P4), while there was no difference between P1 and P4 (Fig. 5a). TG concentration gradually decreased from PHH to ProliHHs (Fig. 5b). Hydroxyproline concentration increased during the dedifferentiation process, however there was no statistical difference (Fig. 5c). These findings indicated the potential biomarkers for cell quality control which is essential in cell therapy.

## Discussion

Previous researches have shown that PHH could gradually dedifferentiated from hepatocyte like to progenitor like state during the ProliHHs proliferating process [15, 16]. A non-invasive and comprehensive quality control method is obligated to link ProliHHs functions with the continuous process, which is critical to apply cell transplantation in clinic [35, 36]. Raman microspectroscopy has superiority to obtain multi- information in a label-free and rapid analysis for different kind of alive cells. Hence, we investigated the feasibility of Raman spectroscopy to capture the cell status in the production of the hepatocytes-derived progenitor like cells at single cell level.

ProliHHs were derived from PHH and passaged four times. The mRNA levels suggested that the mature hepatic function gradually down regulated and progenitor like function up regulated, which was consistent with previous reports [15, 16]. At least 600 Raman spectral were collected for each passage stage. LDA clearly distinguished PHH, ProliHHs P1 and P4. P9 of ProliHHs were also collected and conducted similar analysis (Additional file 1: Fig. S3). It was found that the Raman spectra of ProliHHs P4 and P9 were close but distinguishable, which was consistent with the fact that ProliHHs was more like progenitor cells after P4 [15]. Therefore, P0 (PHH) to P4 of ProliHHs were used as a representative for the following research.

All Raman spectra were sufficiently used to set up a database by machine learning model. A two-layer model was successfully stacked by KNN, LDA, PLS, SVM-Linear, SVM-RBF and RF, the overall accuracy was 84.6%. The results suggested it was feasible to identify ProliHHs status by comparing their Raman bands with database in a few seconds. It was reported that the Raman spectra collected from cell nuclei had the best signal to noise ratio [37]. Therefore, the samples collected in cellular center and peripheral were separately built with machine learning models. The results showed that the overall accuracy was similar (Additional file 2: Table S3). It confirmed the robustness of our established Raman machine learning model, and the location of collection would not affect the prediction results. There is always a balance between robustness and accuracy. When accuracy increases, robustness decreases accordingly [38]. Our model achieved better robustness so that it can tolerate more variability between cell batches. The whole study was conducted twice by ProliHHs from similar donor PHH (Lot: 005, Novabiosis). Another similar study was conducted with different donor PHH (Lot: 201678901, Novabiosis). In all the three independent studies, PHH were able to induce ProliHHs successfully, and the corresponding Raman spectra were collected and analyzed. The machine learning models were able to identify different stages cells with high accuracy. The result was consistent and attached at Additional files 3 and 4, which confirmed the robustness of the model. And other types cells such as 293FT (human embryonic kidneys), HepG2 (human liver hepatocellular carcinoma) and PRH (primary rat hepatocytes) were collected Raman spectroscopy (Additional file 3: Figure S8). The average Raman spectra were significantly different among these cells.

Hepatocytes have various functions such as protein synthesis, biotransformation, energy storage and detoxification. However, its cellular components may change with the cell plasticity during cell dedifferentiation from PHH to ProliHHs [39]. It is not clear if Raman spectra could capture these changes. In this study, a lot of significant changes have been identified within Raman bands.

For example, we have discovered that the mitochondrial associated Raman band at 1003 cm^−1^ (phenylalanine) increased during PHH dedifferentiation. The result suggested that the mitochondria decreased during cell dedifferentiation. In order to verify this, reactive oxygen levels were measured and it was found ROS levels in ProliHHs (P1 and P4) were much lower than PHH (Fig. 5a), which implied the mitochondrial activity was down regulated after PHH was converted to ProliHHs, progenitor-like cells. Actually, it was reported that in order to obtain proliferation, pluripotency and plasticity functions as progenitor-like cells, the reprogramming somatic cells became immature and the amount of mitochondrial DNA was reduced [40–42], which was consistent with our results.

The significant decrease of the Raman band related to lipids were identified at 1080 cm^−1^, 1300 cm^−1^, 1440 cm^−1^, 1744 cm^−1^ respectively. In order to verify the reliability of aforementioned results, triglycerides (TG) concentrations were determined, which was primary lipids storage form in hepatocyte like cells. The result revealed that the TG levels in ProliHHs (P1 and P4) were much lower than PHH (Fig. 5b), which was consistent with Raman measurement. Fu et al. also described that the lipids genes were down regulated when PHH was reprogrammed to liver progenitor like cells [43]. Therefore, the Raman lipids bands may be used as potential hallmarks of hepatocyte-like cell status, or even could be utilized to diagnose non-alcoholic fatty liver disease [44, 45].

Another interesting finding is, with the progress of dedifferentiation to later passages of ProliHHs, the hydroxyproline content significantly increased in Raman spectrum. Our current data revealed the increased hydroxyproline trend in P4 of ProliHHs (Fig. 5c). The up-regulated hydroxyproline may imply that the later passaged ProliHHs activated epithelial-mesenchymal transition (EMT) related pathways. Upon liver injury, mature hepatocytes can be reprogrammed to liver progenitor-like cells (LPLCs) in vivo. Notably, these LPLCs show significant induction of mesenchymal markers as well as progenitor markers, suggesting the activation of EMT pathway during the conversion [39, 46]. A recent single-cell transcriptomics analysis also reveals the emergence of liver progenitors with mesenchymal features during liver development [47]. In line with these in vivo findings, it has been demonstrated that ProliHHs at late passages showed a tendency to express progenitor-like profiles and mesenchymal markers [15]. Therefore, the increased hydroxyproline can possibly be applied as a marker for the conversion of ProliHH to liver progenitors. However, this hypothesis required further verification.

## Conclusion

In short, Raman spectra provided us a lot of information about the changes of biochemical molecules in the process of somatic cells dedifferentiation. To our knowledge, this work is the first report that Raman spectroscopy could successfully identify ProliHHs from P0 to P4. A machine learning model was established at overall accuracy of 84.6%, which made it feasible to conduct real-time ProliHHs quality control for cell transplantation. Raman spectra could capture the change of ROS, hydroxyproline and lipids in hepatocyte-like cells, their changes were consistent with their physiological functions at different cellular status, respectively. Therefore, it is feasible to apply Raman spectra for the identification of progenitor-like cells from somatic cells and obtain multiple cellular components information simultaneously [48].

## Supplementary Information

**Additional file 1: Figure S1.** The location of laser focusing ( +) at the center (A) and periphery (B) in ProliHHs P1 (Lot:005).** Figure S2**. Principal component analysis of all Raman spectra in PHH (Lot:005), ProliHHs P1 and P4 cells. (The red, blue, and green colors represent PHH, ProliHHs P1 and P4 cells, respectively. PHH: primary human hepatocytes, ProliHHs: proliferating human hepatocytes, P1: passage 1, P4: passage 4).** Figure S3**. 10% most significant wavenumbers in LD1 (A) and LD2 (B) contributing to differences among PHH (Lot:005), ProliHHs P1 and P4 cells. (PHH: primary human hepatocytes, ProliHHs: proliferating human hepatocytes, P1: passage 1, P4: passage 4, LD: Linear discriminant).** Figure S4**. LDA analysis of all Raman spectra in PHH (Lot:005), P1, P4 and P9 cells. (LDA: Linear discriminant analysis, PHH: primary human hepatocytes, ProliHHs: proliferating human hepatocytes, P1: passage 1, P4: passage 4, P9: passage 9)

**Additional file 2: Table S1.** Primers for qPCR.** Table S2**. Machine learning model (A) KNN, (B) LDA, (C) PLS, (D) Linear-SVM, (E) RBF-SVM, (F) Random forest to identify cells. Overall accuracy at 62.6%, 67.5%, 63.6%, 83.8%, 81.4%, 65.6%.** Table S3**. Machine learning by stacked (KNN, LDA, PLS, SVM-Linear, SVM-RBF, RF) model to identify cells from center (A) and peripheral (B) Overall accuracy at 83.4%, 82.33%.

**Additional file 3: Figure S5.** Raman spectroscopy and classification analysis for PHH (Lot:201678901), ProliHHs P1 and P4. (A) The averaged spectra (*n* = 1829) collected by PHH (*n* = 619), P1 (*n* = 595) and P4 (*n* = 615) on fingerprint region. (B) Linear discriminant analysis clearly distinguished three cell groups. (The red, blue, and green colors represent PHH, ProliHHs P1 and P4 cells, respectively. PHH: primary human hepatocytes, ProliHHs: proliferating human hepatocytes, P1: passage 1, P4: passage 4).** Figure S6**. The biochemical molecules represented by the specific Raman bands in the average spectral (Lot:201678901).** Figure S7**. The peak area were semi-quantitative to compare differences of the specific Raman bands (A) 480 ${\rm cm}^{-1}$ (glycogen), (B) 831 ${\rm cm}^{-1}$ (tyrosine), (C) 840-860 ${\rm cm}^{-1}$(polysaccharide structure), (D) 1003 ${\rm cm}^{-1}$(phenylalanine), (E) 1080 ${\rm cm}^{-1}$(amide II, typical phospholipid), (F) 1172 ${\rm cm}^{-1}$(C-H in-plane bending mode of tyrosine), (G) 1206 ${\rm cm}^{-1}$(hydroxyproline, tyrosine), (H) 1265 ${\rm cm}^{-1}$(α-helix, collagen, tryptophan), (I) 1300 ${\rm cm}^{-1}$(lipids), (J) 1337 ${\rm cm}^{-1}$(amide III), (K) 1440 ${\rm cm}^{-1}$(lipids), (L) 1658 ${\rm cm}^{-1}$(amide I), (M) 1744 ${\rm cm}^{-1}$(carbonyl feature of lipid spectra) in PHH (Lot:201678901), ProliHHs P1 and P4. The results represent median, ns *p* $\ge$ 0.05, * *p* < 0.05, ** *p* < 0.01, *** *p* < 0.001, **** *p* < 0.0001. (PHH: primary human hepatocytes, ProliHHs: proliferating human hepatocytes, P1: passage 1, P4: passage 4).** Figure S8**. The average Raman spectral of PHH (Lot: 201678901, *n* = 208), PRH (*n* = 201), HepG2 (*n* = 204) and 293FT (*n* = 205). (PHH: primary human hepatocytes, PRH: primary rat hepatocytes, HepG2: human liver hepatocellular carcinoma, 293FT: human embryonic kidneys.).** Table S4** Machine learning by stacked (KNN, LDA, PLS, Linear-SVM, RBF-SVM, RF) model to identify cells. Overall accuracy at 81.32% (Lot: 201678901).

**Additional file 4: Figure S9.** Raman spectroscopy and classification analysis for PHH (Lot:005), ProliHHs P1, P4 and hepatoblast. (A) The averaged spectra (*n* = 815) collected by PHH (*n* = 204), P1 (*n* = 202), P4 (*n* = 202) and hepatoblast (*n* = 207) on fingerprint region. (B) Linear discriminant analysis clearly distinguished three cell groups. (The red, blue, green and purple colors represent PHH, ProliHHs P1, P4 and hepatoblast cells, respectively. PHH: primary human hepatocytes, ProliHHs: proliferating human hepatocytes, P1: passage 1, P4: passage 4).** Figure S10**. The biochemical molecules represented by the specific Raman bands in the average spectral (Lot:005).** Figure S11**. The peak area were semi-quantitative to compare differences of the specific Raman bands (A) 480 ${\mathrm{cm}}^{-1}$ (glycogen), (B) 831 ${\mathrm{cm}}^{-1}$ (tyrosine), (C) 840–860 ${\mathrm{cm}}^{-1}$ (polysaccharide structure), (D) 1003 ${\mathrm{cm}}^{-1}$ (phenylalanine), (E) 1080 ${\mathrm{cm}}^{-1}$ (amide II, typical phospholipid), (F) 1172 ${\mathrm{cm}}^{-1}$ (C–H in-plane bending mode of tyrosine), (G) 1206 ${\mathrm{cm}}^{-1}$ (hydroxyproline, tyrosine), (H) 1265 ${\mathrm{cm}}^{-1}$ (α-helix, collagen, tryptophan), (I) 1300 ${\mathrm{cm}}^{-1}$ (lipids), (J) 1337 ${\mathrm{cm}}^{-1}$ (amide III), (K) 1440 ${\mathrm{cm}}^{-1}$ (lipids), (L) 1658 ${\mathrm{cm}}^{-1}$ (amide I), (M) 1744 ${\mathrm{cm}}^{-1}$ (carbonyl feature of lipid spectra) in PHH (Lot:005), ProliHHs P1, P4 and hepatoblast. The results represent median, ns *p* $\ge$ 0.05, * *p* < 0.05, ** *p* < 0.01, *** *p* < 0.001, **** *p* < 0.0001. (PHH: primary human hepatocytes, ProliHHs: proliferating human hepatocytes, P1: passage 1, P4: passage 4). Table S5 Machine learning by stacked (KNN, LDA, PLS, Linear-SVM, RBF-SVM, RF) model to identify cells. Overall accuracy at 92.08% (Lot: 005).

## Data Availability

The datasets used and/or analysed during the current study are available from the corresponding author on reasonable request.

## Abbreviations

ProliHHs: Proliferating human hepatocytes
PHH: Primary human hepatocytes
LDA: Linear discriminant analysis
ROS: Reactive oxygen species
TG: Triglyceride
NAFLD: Nonalcoholic fatty liver disease
PCA: Principal component analysis
KNN: K-nearest neighbor
PLS: Partial least-squares regression
Linear-SVM: Linear support vector machine
RBF-SVM: Radial basis function kernel support vector machine
RF: Random forest
EMT: Epithelial-mesenchymal transition
LPLCs: Liver progenitor-like cells
