# Supplementary material for: Single cell Raman spectroscopy to identify different stages of proliferating human hepatocytes for cell therapy
Source: Stem Cell Res Ther. 2021 Oct 30;12:555. doi: 10.1186/s13287-021-02619-9 (PMC8556950; doi:10.1186/s13287-021-02619-9)
Supplement: Supplementary file 1 — Additional file 1: Figure S1. The location of laser focusing ( +) at the center (A) and periphery (B) in ProliHHs P1 (Lot:005). Figure S2. Principal component analysis of all Raman spectra in PHH (Lot:005), ProliHHs P1 and P4 cells. (The red, blue, and green colors represent PHH, ProliHHs P1 and P4 cells, respectively. PHH: primary human hepatocytes, ProliHHs: proliferating human hepatocytes, P1: passage 1, P4: passage 4). Figure S3. 10% most significant wavenumbers in LD1 (A) and LD2 (B) contributing to differences among PHH (Lot:005), ProliHHs P1 and P4 cells. (PHH: primary human hepatocytes, ProliHHs: proliferating human hepatocytes, P1: passage 1, P4: passage 4, LD: Linear discriminant). Figure S4. LDA analysis of all Raman spectra in PHH (Lot:005), P1, P4 and P9 cells. (LDA: Linear discriminant analysis, PHH: primary human hepatocytes, ProliHHs: proliferating human hepatocytes, P1: passage 1, P4: passage 4, P9: passage 9) [file 13287_2021_2619_MOESM1_ESM.pdf]

Figure S1

A B

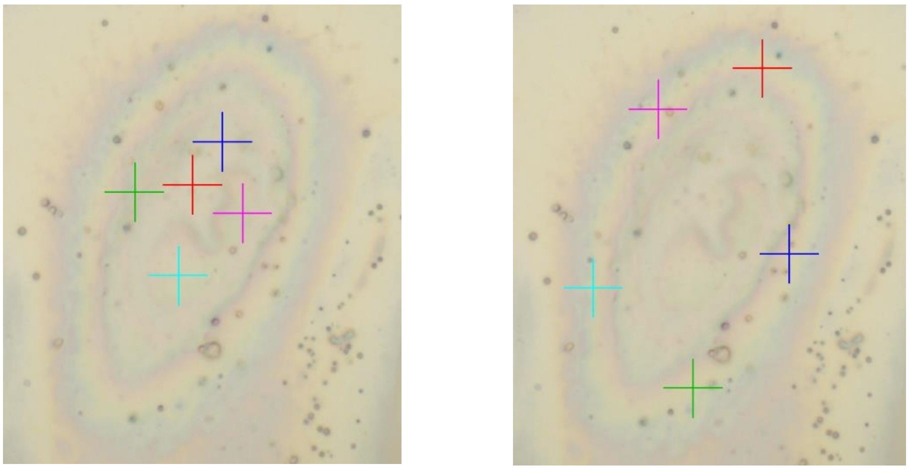

Figure S1. The focusing locations of laser (indicating by crosses with different colors) within the cytoplasm (n=5, A) and on the periphery (n=5, B) based on the brightfield photo of ProliHHs P1 (Lot:005).

Figure S2

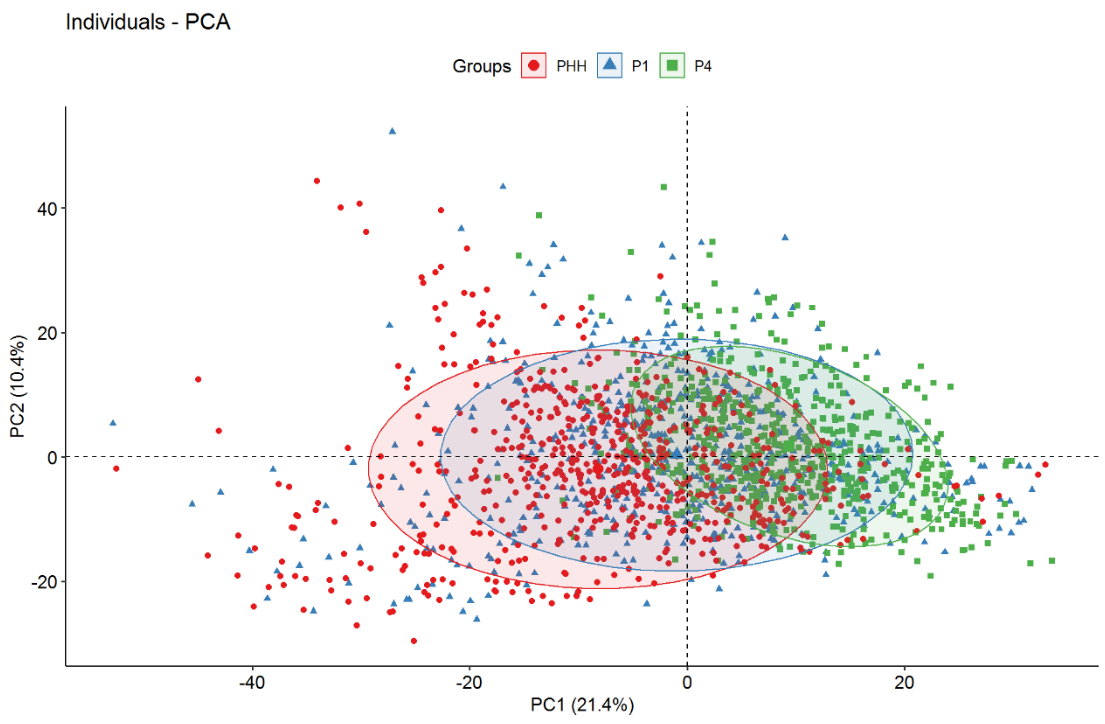

Figure S2. Principal component analysis of all Raman spectra in PHH (Lot:005), ProliHHs P1 and P4 cells. (The red, blue, and green colors represent PHH, ProliHHs P1 and P4 cells, respectively. PHH: primary human hepatocytes, ProliHHs: proliferating human hepatocytes, P1: passage 1, P4: passage 4)

Figure S3

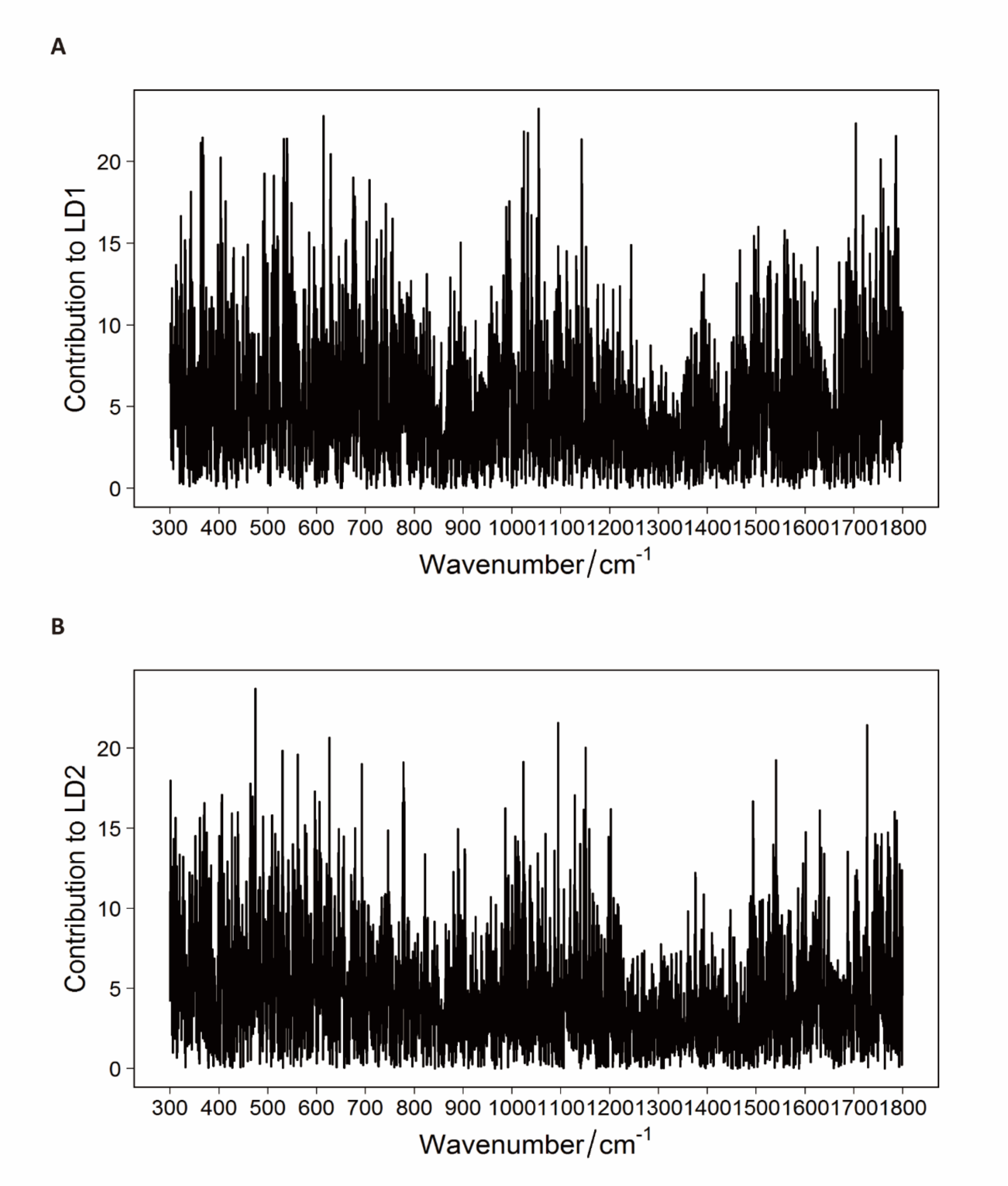

Figure S3. 10% most significant wavenumbers in LD1 (A) and LD2 (B) contributing to differences among PHH (Lot:005), ProliHHs P1 and P4 cells. (PHH: primary human hepatocytes, ProliHHs: proliferating human hepatocytes, P1: passage 1, P4: passage 4, LD: Linear discriminant)

Figure S4

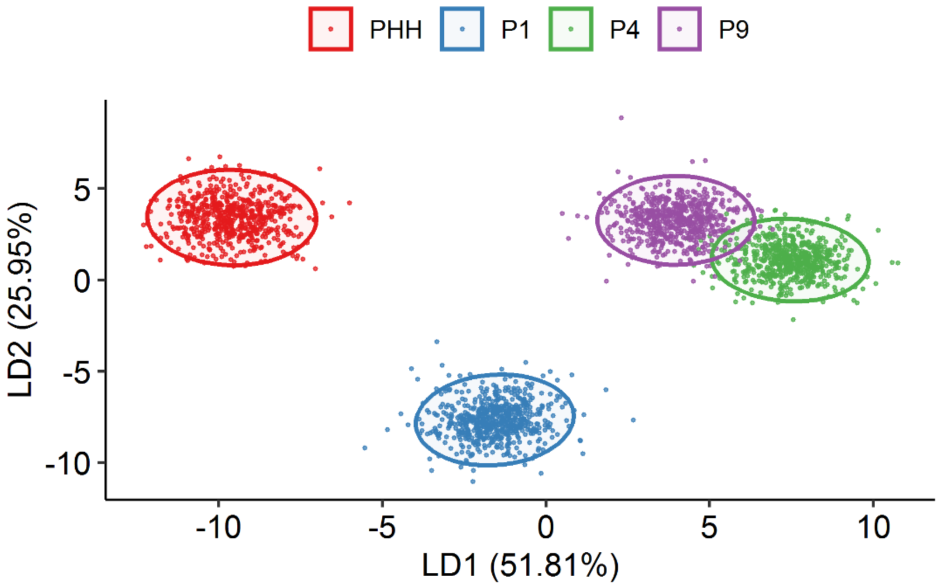

Figure S4. LDA analysis of all Raman spectra in PHH (Lot:005), P1, P4 and P9 cells. (LDA: Linear discriminant analysis, PHH: primary human hepatocytes, ProliHHs: proliferating human hepatocytes, P1: passage 1, P4: passage 4, P9: passage 9)
