# Supplementary material for: Single cell Raman spectroscopy to identify different stages of proliferating human hepatocytes for cell therapy
Source: Stem Cell Res Ther. 2021 Oct 30;12:555. doi: 10.1186/s13287-021-02619-9 (PMC8556950; doi:10.1186/s13287-021-02619-9)
Supplement: Supplementary file 2 — Additional file 2: Table S1. Primers for qPCR. Table S2. Machine learning model (A) KNN, (B) LDA, (C) PLS, (D) Linear-SVM, (E) RBF-SVM, (F) Random forest to identify cells. Overall accuracy at 62.6%, 67.5%, 63.6%, 83.8%, 81.4%, 65.6%. Table S3. Machine learning by stacked (KNN, LDA, PLS, SVM-Linear, SVM-RBF, RF) model to identify cells from center (A) and peripheral (B) Overall accuracy at 83.4%, 82.33%. [file 13287_2021_2619_MOESM2_ESM.docx]

**Supplemental Table:**

Table S1. Primers for qPCR

| Gene name | Direction | Sequence |
| --- | --- | --- |
| GAPDH | Forward | CCACCTTTGACGCTGGG |
|  | Reverse | CATACCAGGAAATGAGCTTGACA |
| SOX9 | Forward | GAGCTCGAAACTGACTGGAAA |
|  | Reverse | CTTCTCTTCTCCTCCTGCAAAG |
| CAR | Forward | GTGCTCCTGTGCGGAGTAG |
|  | Reverse | ATGGCAGATAGGCAGTTTCCC |
| FXR | Forward | AACCATACTCGCAATACAGCAA |
|  | Reverse | ACAGCTCATCCCCTTTGATCC |
| MRP2 | Forward | CCACAAGCCCAGAATAAGGTAG |
|  | Reverse | ACTGACAATTGGTAGGTGAAAGT |
| CYP2B6 | Forward | GCACTCCTCACAGGACTCTTG |
|  | Reverse | CCCAGGTGTACCGTGAAGAC |
| CYP3A4 | Forward | TTCAGCAAGAAGAACAAGGACAA |
|  | Reverse | GGTTGAAGAAGTCCTCCTAAGC |
| UGT1A1 | Forward | CATGCTGGGAAGATACTGTTGAT |
|  | Reverse | GCCCGAGACTAACAAAAGACTCT |
| UGT2B7 | Forward | AAGGTGCTGGTGTGGGCAG |
|  | Reverse | AGCGGATGAGTTGTTGGGA |

Table S2. Machine learning model (A) KNN, (B) LDA, (C) PLS, (D) Linear-SVM, (E) RBF-SVM, (F) Random forest to identify cells. Overall accuracy at 62.6%, 67.5%, 63.6%, 83.8%, 81.4%, 65.6%.

1. KNN: k-nearest neighbor

|  | Reference | | |
| --- | --- | --- | --- |
|  | P1 | P4 | PHH |
| Model prediction |  |  |  |
| P1 | 26 | 6 | 12 |
| P4 | 58 | 131 | 11 |
| PHH | 72 | 14 | 32 |
| Sensitivity(%) | 16.7 | 86.8 | 85.2 |
| Specificity(%) | 94.1 | 77.8 | 72.0 |

PHH: primary human hepatocytes, ProliHHs: proliferating human hepatocytes, P1: passage 1, P4: passage 4

B. LDA: linear discriminant analysis

|  | Reference | | |
| --- | --- | --- | --- |
|  | P1 | P4 | PHH |
| Model prediction |  |  |  |
| P1 | 89 | 27 | 30 |
| P4 | 32 | 106 | 8 |
| PHH | 35 | 18 | 117 |
| Sensitivity(%) | 57.1 | 7.02 | 75.5 |
| Specificity(%) | 81.4 | 87.1 | 82.7 |

PHH: primary human hepatocytes, ProliHHs: proliferating human hepatocytes, P1: passage 1, P4: passage 4

C. PLS: partial least-squares regression

|  | Reference | | |
| --- | --- | --- | --- |
|  | P1 | P4 | PHH |
| Model prediction |  |  |  |
| P1 | 50 | 6 | 33 |
| P4 | 50 | 144 | 22 |
| PHH | 56 | 1 | 100 |
| Sensitivity(%) | 32.1 | 95.4 | 64.5 |
| Specificity(%) | 87.3 | 76.9 | 81.4 |

PHH: primary human hepatocytes, ProliHHs: proliferating human hepatocytes, P1: passage 1, P4: passage 4

D. Linear-SVM: linear support vector machine

|  | Reference | | |
| --- | --- | --- | --- |
|  | P1 | P4 | PHH |
| Model prediction |  |  |  |
| P1 | 116 | 11 | 21 |
| P4 | 17 | 137 | 0 |
| PHH | 23 | 3 | 134 |
| Sensitivity(%) | 74.4 | 90.7 | 86.5 |
| Specificity(%) | 89.5 | 94.5 | 91.5 |

PHH: primary human hepatocytes, ProliHHs: proliferating human hepatocytes, P1: passage 1, P4: passage 4

E. RBF-SVM: radial basis function kernel support vector machine

|  | Reference | | |
| --- | --- | --- | --- |
|  | P1 | P4 | PHH |
| Model prediction |  |  |  |
| P1 | 115 | 10 | 27 |
| P4 | 15 | 137 | 4 |
| PHH | 26 | 4 | 124 |
| Sensitivity(%) | 73.7 | 90.7 | 80.0 |
| Specificity(%) | 87.9 | 93.9 | 90.2 |

PHH: primary human hepatocytes, ProliHHs: proliferating human hepatocytes, P1: passage 1, P4: passage 4

F. RF: random forest

|  | Reference | | |
| --- | --- | --- | --- |
|  | P1 | P4 | PHH |
| Model prediction |  |  |  |
| P1 | 67 | 16 | 34 |
| P4 | 42 | 128 | 13 |
| PHH | 47 | 7 | 108 |
| Sensitivity(%) | 43.0 | 84.8 | 69.7 |
| Specificity(%) | 83.7 | 82.3 | 82.4 |

PHH: primary human hepatocytes, ProliHHs: proliferating human hepatocytes, P1: passage 1, P4: passage 4

Table S3. Machine learning by stacked (KNN, LDA, PLS, SVM-Linear, SVM-RBF, RF) model to identify cells from center (A) and peripheral (B). Overall accuracy at 83.4%, 82.33%.

A. center

|  | Reference | | |
| --- | --- | --- | --- |
|  | P1 | P4 | PHH |
| Model prediction |  |  |  |
| P1 | 71 | 7 | 13 |
| P4 | 11 | 68 | 1 |
| PHH | 7 | 1 | 62 |
| Sensitivity(%) | 79.78 | 89.47 | 81.58 |
| Specificity(%) | 86.84 | 92.73 | 95.15 |

KNN: k-nearest neighbor, LDA: linear discriminant analysis, PLS: partial least-squares regression, Linear-SVM: linear support vector machine, RBF-SVM: radial basis function kernel support vector machine, RF: random forest, PHH: primary human hepatocytes, ProliHHs: proliferating human hepatocytes, P1: passage 1, P4: passage 4

B. peripheral

|  | Reference | | |
| --- | --- | --- | --- |
|  | P1 | P4 | PHH |
| Model prediction |  |  |  |
| P1 | 44 | 5 | 11 |
| P4 | 13 | 68 | 1 |
| PHH | 8 | 0 | 65 |
| Sensitivity(%) | 67.69 | 93.15 | 84.42 |
| Specificity(%) | 89.33 | 90.14 | 94.2 |

KNN: k-nearest neighbor, LDA: linear discriminant analysis, PLS: partial least-squares regression, Linear-SVM: linear support vector machine, RBF-SVM: radial basis function kernel support vector machine, RF: random forest, PHH: primary human hepatocytes, ProliHHs: proliferating human hepatocytes, P1: passage 1, P4: passage 4
