# Supplementary material for: Single cell Raman spectroscopy to identify different stages of proliferating human hepatocytes for cell therapy
Source: Stem Cell Res Ther. 2021 Oct 30;12:555. doi: 10.1186/s13287-021-02619-9 (PMC8556950; doi:10.1186/s13287-021-02619-9)
Supplement: Supplementary file 3 — Additional file 3: Figure S5. Raman spectroscopy and classification analysis for PHH (Lot:201678901), ProliHHs P1 and P4. (A) The averaged spectra (n = 1829) collected by PHH (n = 619), P1 (n = 595) and P4 (n = 615) on fingerprint region. (B) Linear discriminant analysis clearly distinguished three cell groups. (The red, blue, and green colors represent PHH, ProliHHs P1 and P4 cells, respectively. PHH: primary human hepatocytes, ProliHHs: proliferating human hepatocytes, P1: passage 1, P4: passage 4). Figure S6. The biochemical molecules represented by the specific Raman bands in the average spectral (Lot:201678901). Figure S7. The peak area were semi-quantitative to compare differences of the specific Raman bands (A) 480 ${\rm cm}^{-1}$cm-1 (glycogen), (B) 831 ${\rm cm}^{-1}$cm-1 (tyrosine), (C) 840-860 ${\rm cm}^{-1}$cm-1(polysaccharide structure), (D) 1003 ${\rm cm}^{-1}$cm-1(phenylalanine), (E) 1080 [file 13287_2021_2619_MOESM3_ESM.pdf]

Figure S5

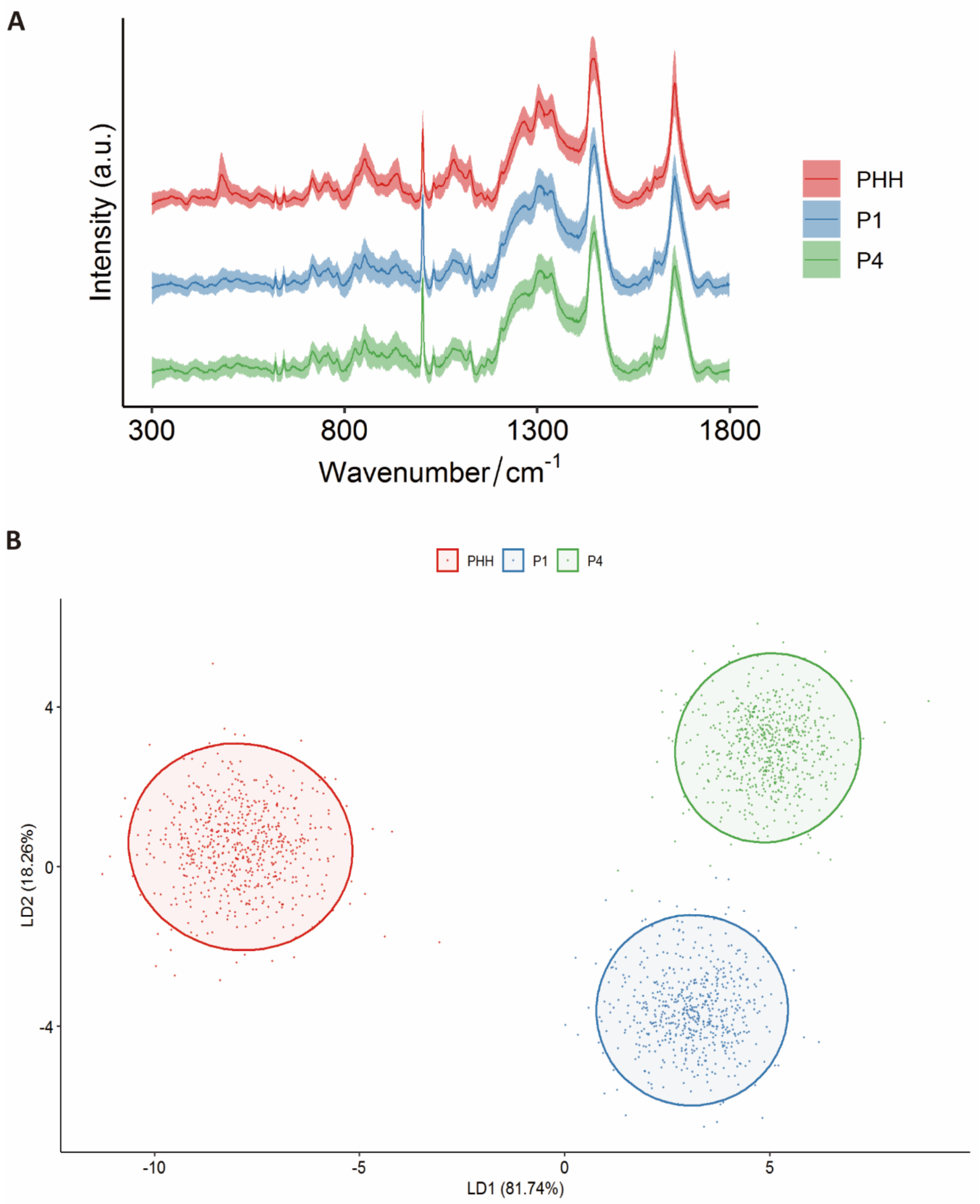

Figure S5. Raman spectroscopy and classification analysis for PHH (Lot:201678901), ProliHHs P1 and P4. (A) The averaged spectra (n = 1829) collected by PHH (n = 619), P1 (n = 595) and P4 (n = 615) on fingerprint region. (B) Linear discriminant analysis clearly distinguished three cell groups. (The red, blue, and green colors represent PHH, ProliHHs P1 and P4 cells, respectively. PHH: primary human hepatocytes, ProliHHs: proliferating human hepatocytes, P1: passage 1, P4: passage 4)

Figure S6

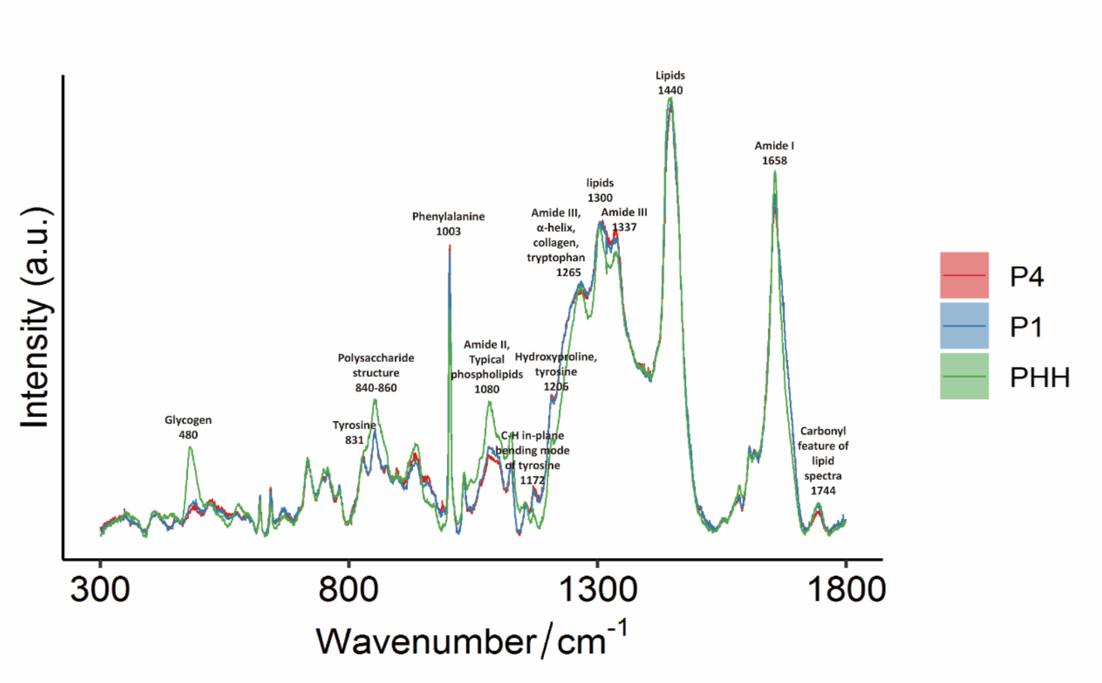

Figure S6. The biochemical molecules represented by the specific Raman bands in the average spectral (Lot:201678901).

Figure S7

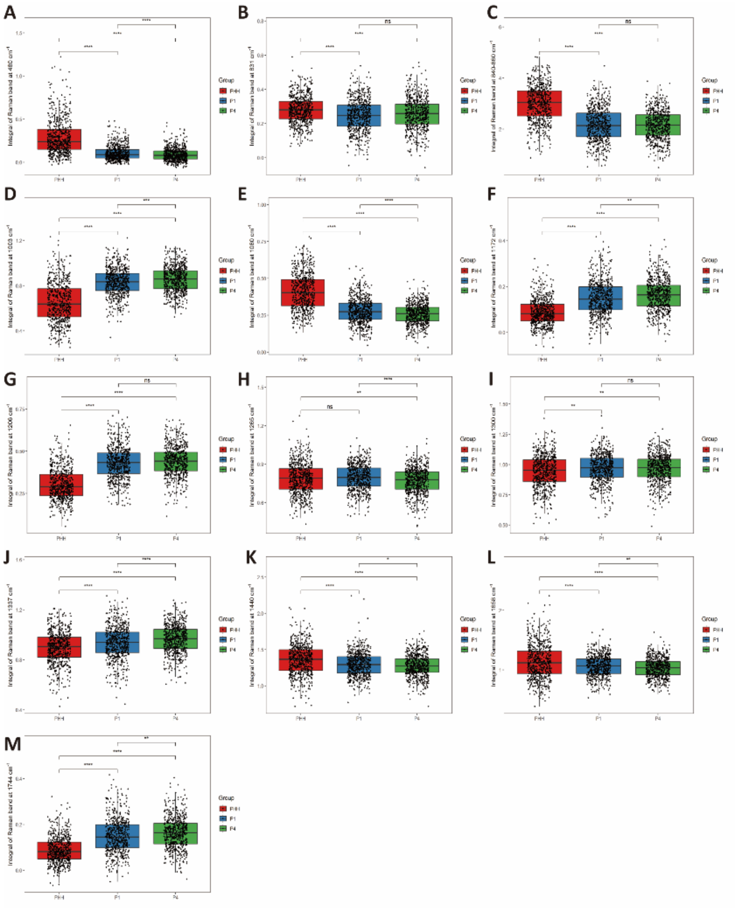

Figure S7. The peak area were semi-quantitative to compare differences of the specific Raman bands (A) 480 cm^{-1} (glycogen), (B) 831 cm^{-1} (tyrosine), (C) 840-860 cm^{-1} (polysaccharide structure), (D) 1003 cm^{-1} (phenylalanine), (E) 1080 cm^{-1} (amide II, typical phospholipid), (F) 1172 cm^{-1} (C-H in-plane bending mode of tyrosine), (G) 1206 cm^{-1} (hydroxyproline, tyrosine), (H) 1265 cm^{-1} (α-helix, collagen, tryptophan), (I) 1300 cm^{-1} (lipids), (J) 1337 cm^{-1}(amide III), (K) 1440 cm^{-1}(lipids), (L) 1658 cm^{-1} (amide I), (M) 1744 cm^{-1} (carbonyl feature of lipid spectra) in PHH (Lot:201678901), ProliHHs P1 and P4. The results represent median, ns $P \geq 0.05$, * $P < 0.05$, ** $P < 0.01$, *** $P < 0.001$, **** $P < 0.0001$. (PHH: primary human hepatocytes, ProliHHs: proliferating human hepatocytes, P1: passage 1, P4: passage 4)

Figure S8

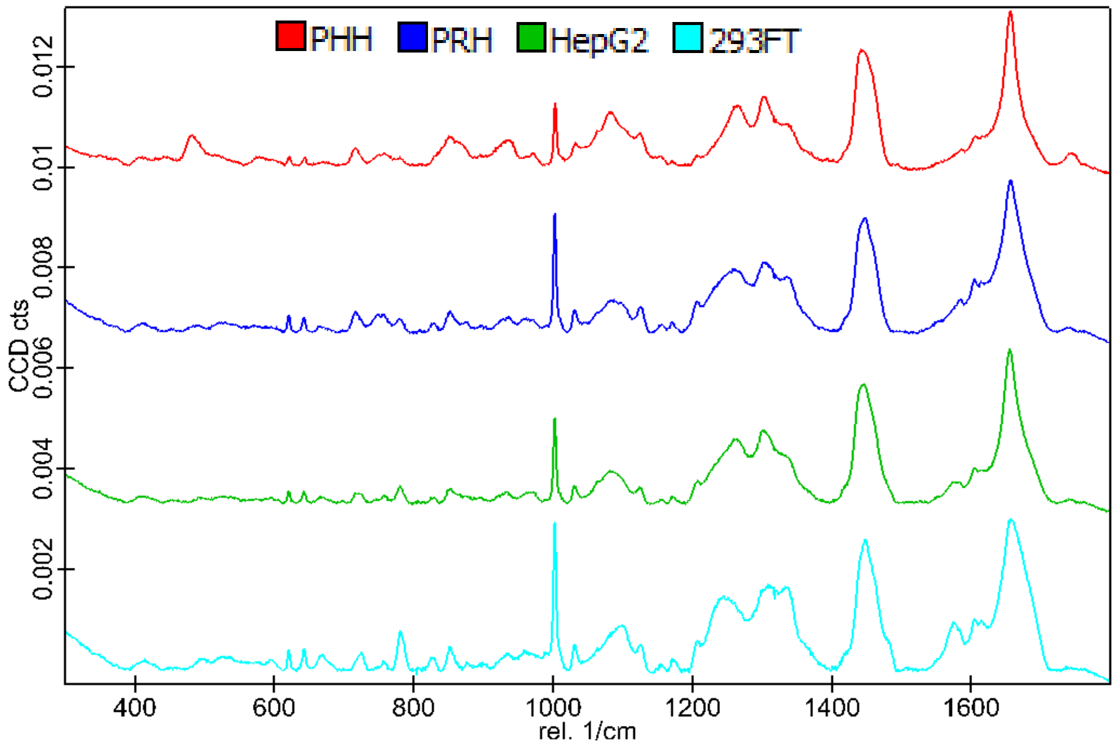

Figure S8. The average Raman spectral of PHH (Lot: 201678901, n=208), PRH (n=201), HepG2 (n=204) and 293FT (n=205). (PHH: primary human hepatocytes, PRH: primary rat hepatocytes, HepG2: human liver hepatocellular carcinoma, 293FT: human embryonic kidneys.)

Table S4 Machine learning by stacked (KNN, LDA, PLS, Linear-SVM, RBF-SVM, RF) model to identify cells. Overall accuracy at 81.32% (Lot: 201678901).

	Reference		
	P1	P4	PHH
Model prediction			
P1	106	33	7
P4	38	118	1
PHH	4	2	146
Sensitivity(%)	71.62	77.12	94.81
Specificity(%)	86.97	87.09	98.01
