# Supplementary material for: Single cell Raman spectroscopy to identify different stages of proliferating human hepatocytes for cell therapy
Source: Stem Cell Res Ther. 2021 Oct 30;12:555. doi: 10.1186/s13287-021-02619-9 (PMC8556950; doi:10.1186/s13287-021-02619-9)
Supplement: Supplementary file 4 — Additional file 4: Figure S9. Raman spectroscopy and classification analysis for PHH (Lot:005), ProliHHs P1, P4 and hepatoblast. (A) The averaged spectra (n = 815) collected by PHH (n = 204), P1 (n = 202), P4 (n = 202) and hepatoblast (n = 207) on fingerprint region. (B) Linear discriminant analysis clearly distinguished three cell groups. (The red, blue, green and purple colors represent PHH, ProliHHs P1, P4 and hepatoblast cells, respectively. PHH: primary human hepatocytes, ProliHHs: proliferating human hepatocytes, P1: passage 1, P4: passage 4). Figure S10. The biochemical molecules represented by the specific Raman bands in the average spectral (Lot:005). Figure S11. The peak area were semi-quantitative to compare differences of the specific Raman bands (A) 480 ${\mathrm{cm}}^{-1}$cm-1 (glycogen), (B) 831 ${\mathrm{cm}}^{-1}$cm-1 (tyrosine), (C) 840–860 ${\mathrm{cm}}^{-1}$cm-1 (polysaccharide structure), (D) 1003 ${\mathrm{cm}}^{-1}$cm-1 (phenylalanine), (E) 1080 [file 13287_2021_2619_MOESM4_ESM.pdf]

Figure S9

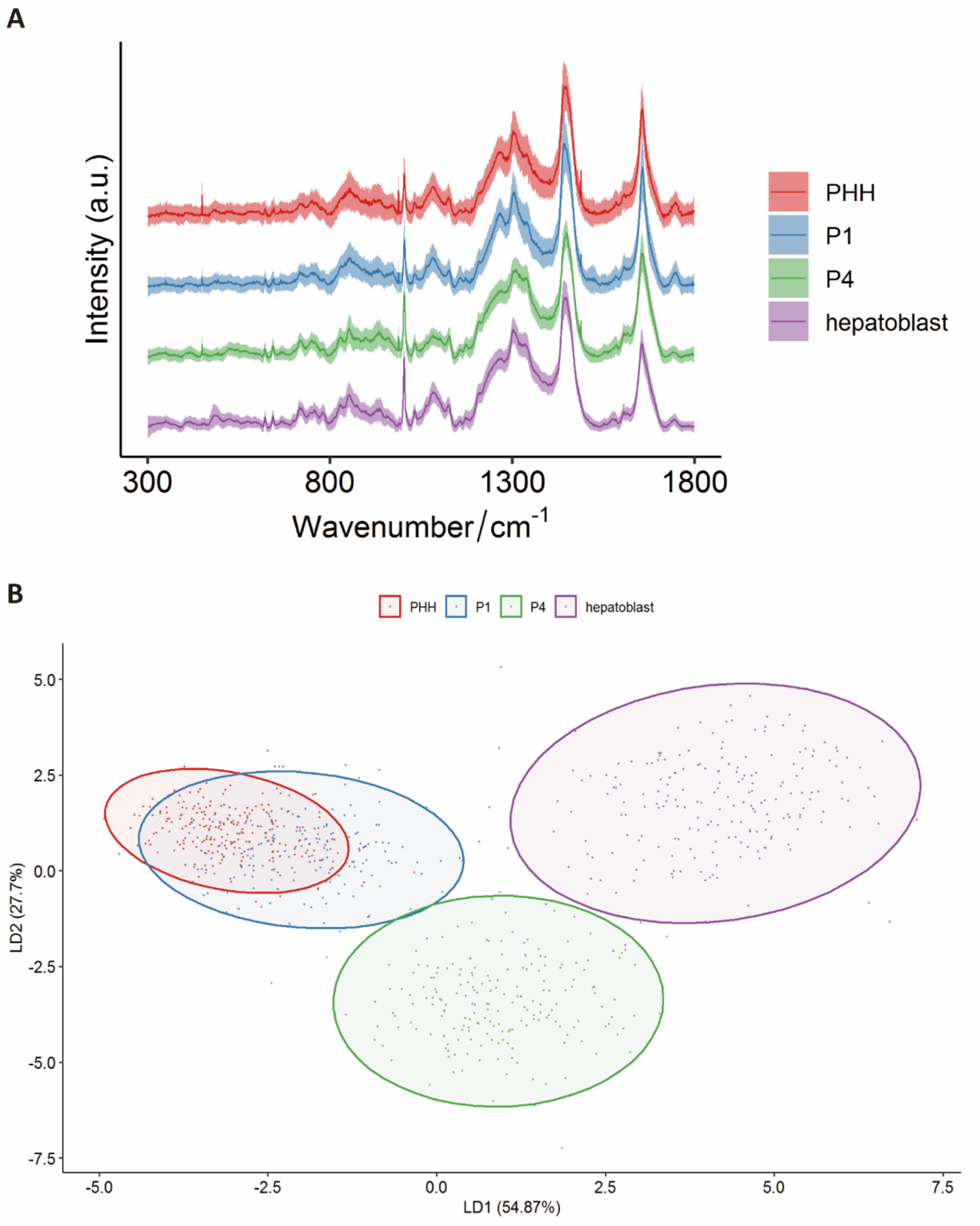

Figure S9. Raman spectroscopy and classification analysis for PHH (Lot:005), ProliHHs P1, P4 and hepatoblast. (A) The averaged spectra (n = 815) collected by PHH (n = 204), P1 (n = 202), P4 (n = 202) and hepatoblast (n=207) on fingerprint region. (B) Linear discriminant analysis clearly distinguished three cell groups. (The red, blue, green and purple colors represent PHH, ProliHHs P1, P4 and hepatoblast cells, respectively. PHH: primary human hepatocytes, ProliHHs: proliferating human hepatocytes, P1: passage 1, P4: passage 4)

Figure S10

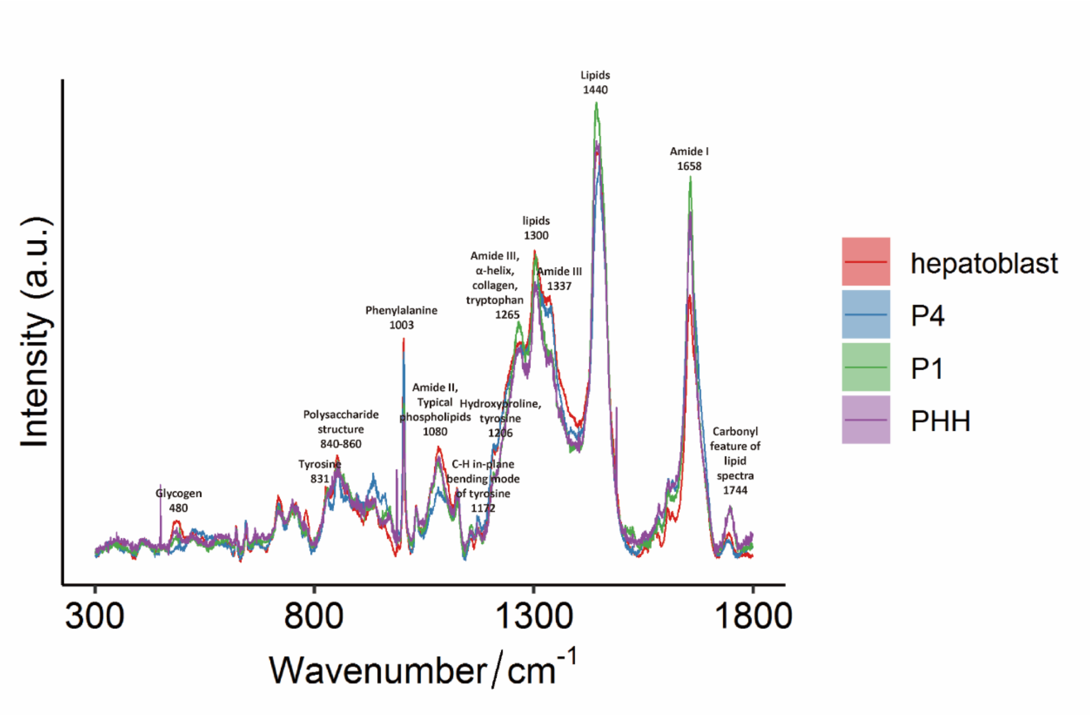

Figure S10. The biochemical molecules represented by the specific Raman bands in the average spectral (Lot:005).

Figure S11

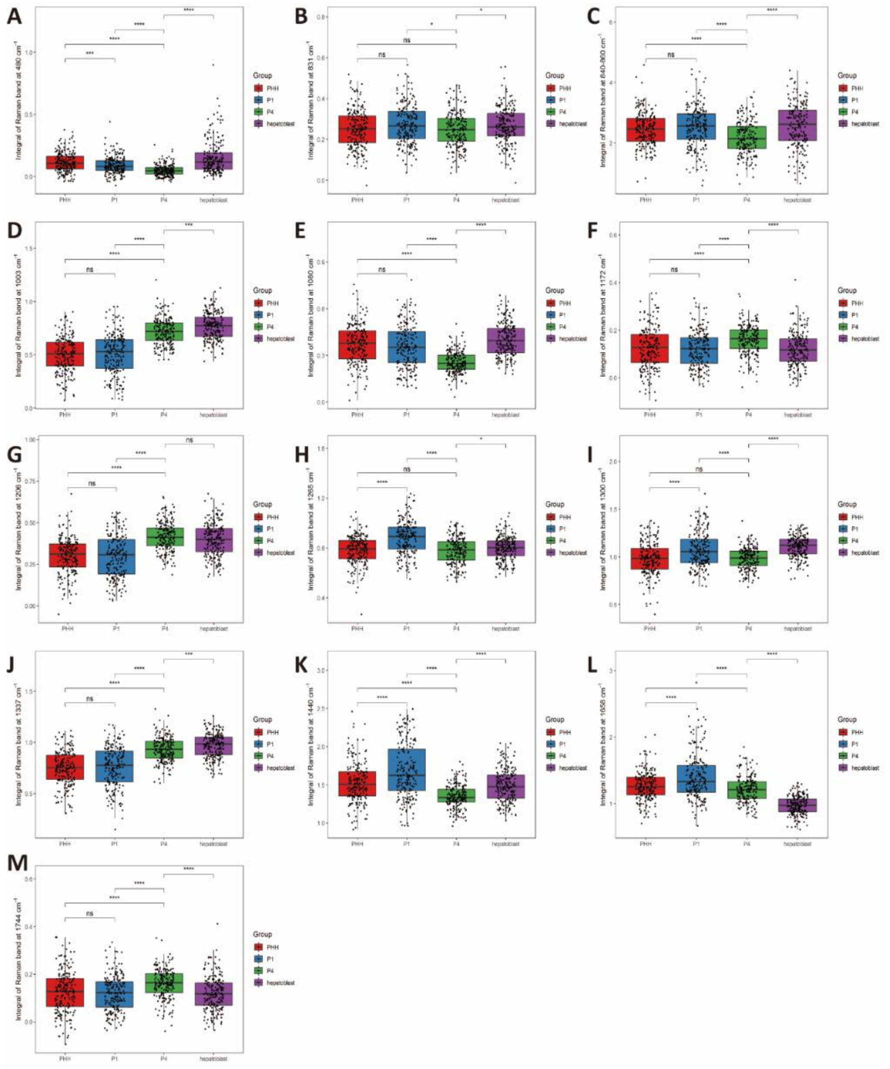

Figure S11. The peak area were semi-quantitative to compare differences of the specific Raman bands (A) 480 cm^{-1} (glycogen), (B) 831 cm^{-1} (tyrosine), (C) 840-860 cm^{-1} (polysaccharide structure), (D) 1003 cm^{-1} (phenylalanine), (E) 1080 cm^{-1} (amide II, typical phospholipid), (F) 1172 cm^{-1} (C-H in-plane bending mode of tyrosine), (G) 1206 cm^{-1} (hydroxyproline, tyrosine), (H) 1265 cm^{-1} (α-helix, collagen, tryptophan), (I) 1300 cm^{-1} (lipids), (J) 1337 cm^{-1}(amide III), (K) 1440 cm^{-1}(lipids), (L) 1658 cm^{-1} (amide I), (M) 1744 cm^{-1} (carbonyl feature of lipid spectra) in PHH (Lot:005), ProliHHs P1, P4 and hepatoblast. The results represent median, ns P≥0.05, * $P < 0.05$, ** $P < 0.01$, *** $P < 0.001$, **** $P < 0.0001$. (PHH: primary human hepatocytes, ProliHHs: proliferating human hepatocytes, P1: passage 1, P4: passage 4)

Table S5 Machine learning by stacked (KNN, LDA, PLS, Linear-SVM, RBF-SVM, RF) model to identify cells. Overall accuracy at 92.08% (Lot: 005).

	Reference			
	hepatoblast	P1	P4	PHH
Model prediction				
hepatoblast	49	1	0	0
P1	1	45	0	7
P4	1	1	49	1
PHH	0	3	1	43
Sensitivity(%)	96.08	90	98	84.31
Specificity(%)	99.34	94.74	98.03	97.35
